# Modifications of diketopiperazines assembled by cyclodipeptide synthases with cytochrome P_450_ enzymes

**DOI:** 10.1007/s00253-021-11178-1

**Published:** 2021-02-24

**Authors:** Lauritz Harken, Shu-Ming Li

**Affiliations:** Institut für Pharmazeutische Biologie und Biotechnologie, Fachbereich Pharmazie, Philipps-Universität Marburg, Robert-Koch-Str. 4, 35037 Marburg, Germany

**Keywords:** Cyclodipeptides, Cytochrome P450, Diversity of cyclodipeptides, Enzymatic modification

## Abstract

2,5-Diketopiperazines are the smallest cyclic peptides comprising two amino acids connected *via* two peptide bonds. They can be biosynthesized in nature by two different enzyme families, either by nonribosomal peptide synthetases or by cyclodipeptide synthases. Due to the stable scaffold of the diketopiperazine ring, they can serve as precursors for further modifications by different tailoring enzymes, such as methyltransferases, prenyltransferases, oxidoreductases like cyclodipeptide oxidases, 2-oxoglutarate-dependent monooxygenases and cytochrome P_450_ enzymes, leading to the formation of intriguing secondary metabolites. Among them, cyclodipeptide synthase-associated P_450_s attracted recently significant attention, since they are able to catalyse a broader variety of astonishing reactions than just oxidation by insertion of an oxygen. The P_450_-catalysed reactions include hydroxylation at a tertiary carbon, aromatisation of the diketopiperazine ring, intramolecular and intermolecular carbon-carbon and carbon-nitrogen bond formation of cyclodipeptides and nucleobase transfer reactions. Elucidation of the crystal structures of three P_450_s as cyclodipeptide dimerases provides a structural basis for understanding the reaction mechanism and generating new enzymes by protein engineering. This review summarises recent publications on cyclodipeptide modifications by P_450_s.

**Key Points**

*• Intriguing reactions catalysed by cyclodipeptide synthase-associated cytochrome P*_*450*_*s*

*• Homo- and heterodimerisation of diketopiperazines*

*• Coupling of guanine and hypoxanthine with diketopiperazines*

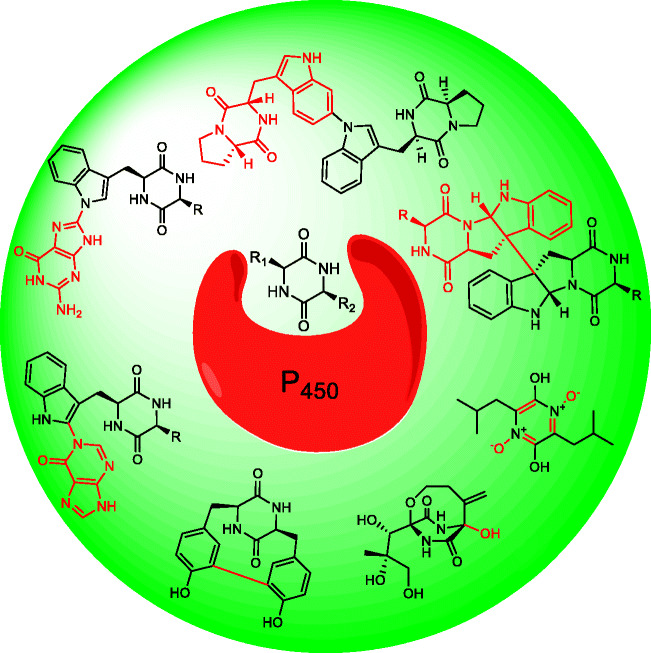

## Introduction

Natural products derived from microbial, plant or animal organisms constitute the largest source for medicinal drugs, either in unmodified form or as chemically modified derivatives (Newman and Cragg 2020). Mining of microbial genome sequences has strongly accelerated the elucidation process of biosynthetic pathways of known metabolites and revealed the presence of a large number of unknown biosynthetic gene clusters (BGCs). They are responsible for new extraordinary enzymes and an astonishing variety of secondary metabolites. As these BGCs are usually silent and their original triggers are diverse, unspecific or even hazardous, often only a targeted activation either in the natural host or in optimised expression hosts provides insights into their functions. The characterised secondary metabolite enzymes are able to catalyse stereoselective, stereospecific, efficient and energetically disfavoured reactions.

**Fig. 1:**
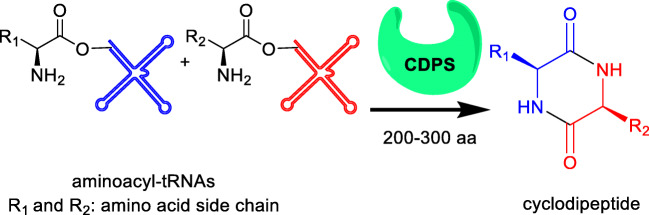
Cyclodipeptide formation catalysed by cyclodipeptide synthases with aminoacyl-tRNAs as substrates

Cyclodipeptides (CDPs) are the smallest possible cyclic peptides from two amino acids with two peptide bonds. In nature, CDPs are assembled by either nonribosomal peptide synthetases (NRPSs) mostly in fungi or by cyclodipeptide synthases (CDPSs) mainly in bacteria. These two enzyme families differ not only in protein size and sequences but also in substrates and reaction mechanisms. NRPSs are large multi-modular proteins using free amino acids as substrates (Izoré and Cryle 2018; Payne et al. 2017). Diketopiperazine (DKP)-forming NRPSs are dimodular enzymes with typical peptide chain lengths of about 2300–2500 amino acids (Xu et al. 2014). In comparison, CDPSs consist only of 200–300 amino acids and hijack the activated aminoacyl-tRNAs from the ribosomal machinery for CDP formation (Fig. 1) (Gondry et al. 2009, 2018; Moutiez et al. 2017).

## Modification of CDPs by tailoring enzymes

The DKP ring of the CDPs has an increased stability against proteolysis, in comparison to acyclic dipeptides (Borthwick 2012), making it a stable scaffold for diverse modifications by tailoring enzymes such as oxidoreductases including 2-oxoglutarate-dependent monooxygenases, cyclodipeptide oxidases (CDOs) and cytochrome P_450_ enzymes (P_450_s), methyltransferases (MTs) and prenyltransferases (Borgman et al. 2019; Canu et al. 2020). Known CDP modification reactions by CDPS-associated tailoring enzymes are listed in Table 1. The products of CDPS-related pathways exhibit diverse pharmacological effects such as antibiotic (Cain et al. 2003), antifungal (Musetti et al. 2007; Ström et al. 2002), anti-inflammatory (Minelli et al. 2012), immunosuppressive (Waring and Beaver 1996) and antitumor activities (Yamazaki et al. 2012). Bicyclomycin, for example, is used as an antibiotic for treatment of traveller’s diarrhoea caused by gram-negative bacteria such as *E. coli*, *Klebsiella*, *Shigella* or *Salmonella* species. It has a unique pharmacological mechanism by selectively inhibiting the bacterial transcription termination factor *Rho* and shows synergetic effects with other antibiotics (Kohn and Widger 2005; Lawson et al. 2016). Mycocyclosin is essential for the viability of *Mycobacterium tuberculosis*. The P_450_ enzyme CYP121 involved in its formation could be therefore considered an alternative target for potential drugs in the treatment of tuberculosis, which is still responsible for 1.5 million deaths worldwide per year (Harding 2020; McLean et al. 2008).

**Table 1:** Overview on enzymatic modifications of cyclodipeptides assembled by cyclodipeptide synthases

Modification by tailoring enzymes	Organism	Reference
a,β-dehydrogenation by cyclodipeptide oxidase	*Streptomyces noursei* and related species *Nocardiopsis dassonvillei, N. alba, N. prasina*	(Giessen et al. 2013b; Lautru et al. 2002; Le Chevalier et al. 2020; Mikulski et al. 2020; Zhang et al. 2013)
N-methylation at DKP ring by methyltransferase	*Actinosynnema mirum* *S. youssoufiensis* and related species	(Giessen et al. 2013a; Yao et al. 2020)
Prenylation by prenyltransferase	*Streptomyces youssoufiensis*	(Yao et al. 2018)
Hydroxylation by 2-oxoglutarate dependent oxygenase and P_450_	*Streptomyces cinnamoneus, Pseudomonas aeruginosa*	(Meng et al. 2018; Patteson et al. 2017; Vior et al. 2018; Witwinowski et al. 2019)
Aromatisation by P_450_	*Bacillus subtilis*	(Cryle et al. 2010)
Intramolecular C-C bond formation by P_450_	*Mycobacterium tuberculosis*	(Belin et al. 2009)
Cyclodipeptide dimerisation by P_450_	*Streptomyces species, Saccharopolyspora antimicrobica*	(Liu et al. 2020; Shende et al. 2020; Sun et al. 2020; Tian et al. 2018; Yu and Li 2019)
Nucleobase addition by P_450_	*Streptomyces purpureus, S. varsoviensis, S. monomycini* S. *lavendulae* and S. *xanthophaeus*	(Liu et al. 2019; Shi et al. 2019; Yu et al. 2018, 2019)

**Fig. 2:**
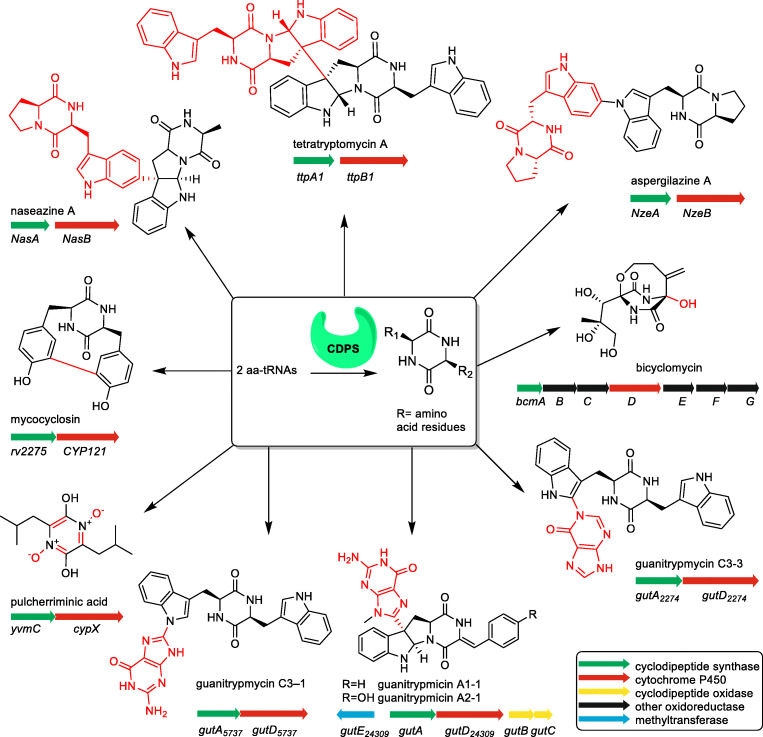
Examples of modification reactions catalysed by CDPS-associated P450s (highlighted in red)

Several outstanding reviews have already outlined the variety of cyclodipeptides and their derivatives (Borgman et al. 2019; Canu et al. 2020; Giessen and Marahiel 2015; Moutiez et al. 2017). During the last years, CDPS-associated P_450_s got more and more attraction. Seventeen members from this enzyme family have been proven to catalyse intriguing reactions. Their catalytic spectrum ranges from hydroxylation of a tertiary carbon, aromatisation, intramolecular C-C bond formation and DKP dimerisation, to transfer of nucleobases to a DKP unit, as exemplarily given in Fig. 2.

## Properties of P_450_s

Cytochrome P_450_ enzymes represent an enzyme superfamily occurring almost ubiquitously throughout living organisms. In eukaryotes, they are usually bound to membranes, whereas bacterial members appear freely in the cytosol. They contribute a major part in carbon source assimilation, production of secondary metabolites and metabolism of xenobiotics (Chen et al. 2021). P_450_s have different sizes, cofactors and electron donors as well as various shapes of substrate binding pockets. These features make them versatile enzymes performing a vast variety of stereoselective and stereospecific reactions. The enzymes of this family got their names based on the Soret peak at 450 nm, when the reduced form is complexed with carbon monoxide (Klingenberg 1957). P_450_s contain a haem acting as a monooxygenase by usually inserting or adding a single oxygen atom onto their substrates. Haem itself is a hexa-coordinated complex consisting of an iron ion in the middle of a planar porphyrin as tetradentate ligand. The iron ion is axially bound to the enzyme on one side and on the opposite side complexed with a molecule water in the resting state. Replacing the water molecule by a reactive oxygen species initiates the P_450_-catalysed reaction. The central iron ion state is changed from Fe^IV^ to Fe^II^ (Katagiri et al. 1968).

Bacterial P_450_s are normally soluble proteins with around 400–500 amino acid residues and can be overproduced in *E. coli* for *in vitro* assays. They use usually ferredoxin and ferredoxin reductase as cofactors but are also able to accept electrons from heterologous redox partners (Rudolf et al. 2017).

## P450 as hydroxylase in the biosynthesis of bicyclomycin

**Fig. 3:**
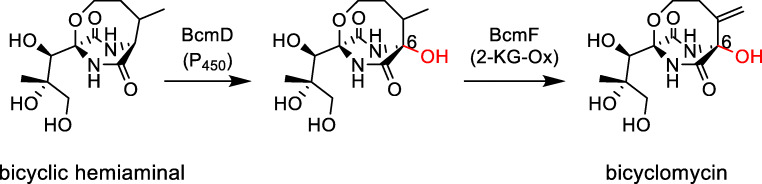
P_450_ role in the biosynthesis of bicyclomycin in *Streptomyces cinnamoneus*

In the BGC of bicyclomycin from *Streptomyces sapporensis*, the P_450_ BcmD acts as a hydroxylase (Fig. 3). The elucidation of the biosynthetic pathway was reported by two different groups in *Streptomyces cinnamoneus* ATCC 21532 (synonym *Streptomyces sapporensis*) (Meng et al. 2018; Patteson et al. 2017). A very similar BGC coding for bicyclomycin biosynthetic pathway was found in *Pseudomonas aeruginosa* SCV20265 (Vior et al. 2018). Several putative bicyclomycin BGCs have also been found in various gram-positive and gram-negative bacteria, suggesting gene transfer events across different bacterial species (Vior et al. 2018).

The CDP core of bicyclomycin consisting of l-isoleucine and l-leucine is modified by five non-haem mononuclear iron and 2-ketoglutarate-dependent oxidases and one P_450_. The bicyclic hemiaminal intermediate is hydroxylated by the P_450_ BcmD and dehydrogenated by the 2-ketoglutarate-dependent oxidase BcmF (Fig. 3).

## P450 as aromatase in the biosynthesis of pulcherriminic acid

**Fig. 4:**
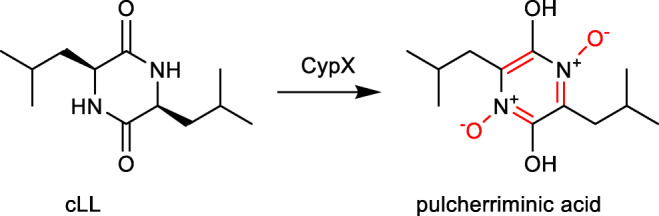
P_450_-catalysed formation of pulcherriminic acid in *Bacillus subtilis*

Pulcherriminic acid has already been isolated and identified in 1972 from *Bacillus subtilis* (Uffen and Canale-Parola 1972), whereas its biosynthetic pathway was elucidated 38 years later (Cryle et al. 2010). The BGC of pulcherriminic acid comprises merely two genes coding for a CPDS and a P_450_ enzyme. The CDP core is assembled from two l-leucine molecules by the CDPS YvmC and altered by the P450 CypX (also termed as CYP134A1) *via* a three-step oxidative transfer mechanism. The two nitrogen atoms of the DKP ring are oxidised to N-oxides and the DKP ring is aromatised (Fig. 4). This aromatisation is claimed either *via* hydroxylation and subsequent water elimination or *via* a direct electron transfer reaction. Afterwards, the oxygen residues undergo a chelation with 2x Fe^3+^ forming pulcherrimin.

## P_450_ catalysing intramolecular C-C bond formation in the biosynthesis of mycocyclosin

**Fig. 5:**
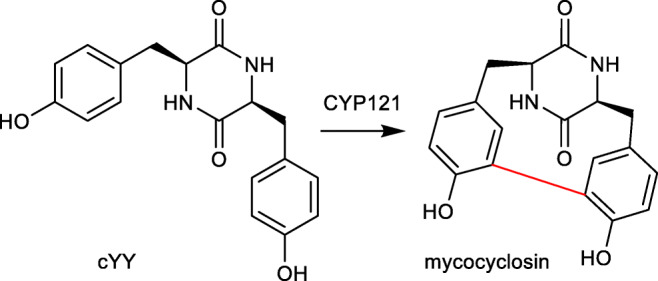
Conversion of cYY to mycocyclosin by the P_450_ enzyme CYP121 in *Mycobacterium tuberculosis*

Most sequenced *Mycobacterium tuberculosis* strains share a two-gene BGC being responsible for the biosynthesis of mycocyclosin, an oxidised cYY product. The CDPS Rv2275 catalyses the condensation of two l-tyrosine molecules to *cyclo*-(l-Tyr-l-Tyr), which is then converted to mycocyclosin by the P_450_ enzyme Rv2276, commonly known as CYP121 (Belin et al. 2009). This conversion is an intramolecular C-C bond formation between the *ortho*-positions to the phenolic residues (Fig. 5). CYP121 shares high sequence homology with P_450_s catalysing nucleobase transfer reactions (Yu et al. 2018).

## P_450_s as transferases of nucleobases guanine and hypoxanthine

Tryptophan is a frequent target of bacterial tailoring enzymes due to its electron-rich indole moiety (Alkhalaf and Ryan 2015). Very recently, bacterial P_450_s from several *Streptomyces* species have been proven to catalyse the linkage of a guanine to a tryptophan residue of CDPs in the biosynthesis of guanitrypmycins and analogues (Liu et al. 2019; Shi et al. 2019; Yu et al. 2018). The elucidation of the biosynthetic pathways was achieved by heterologous expression in *Streptomyces coelicolor*, precursor feeding experiments and biochemical characterisation with recombinant and purified enzymes.

**Fig. 6:**
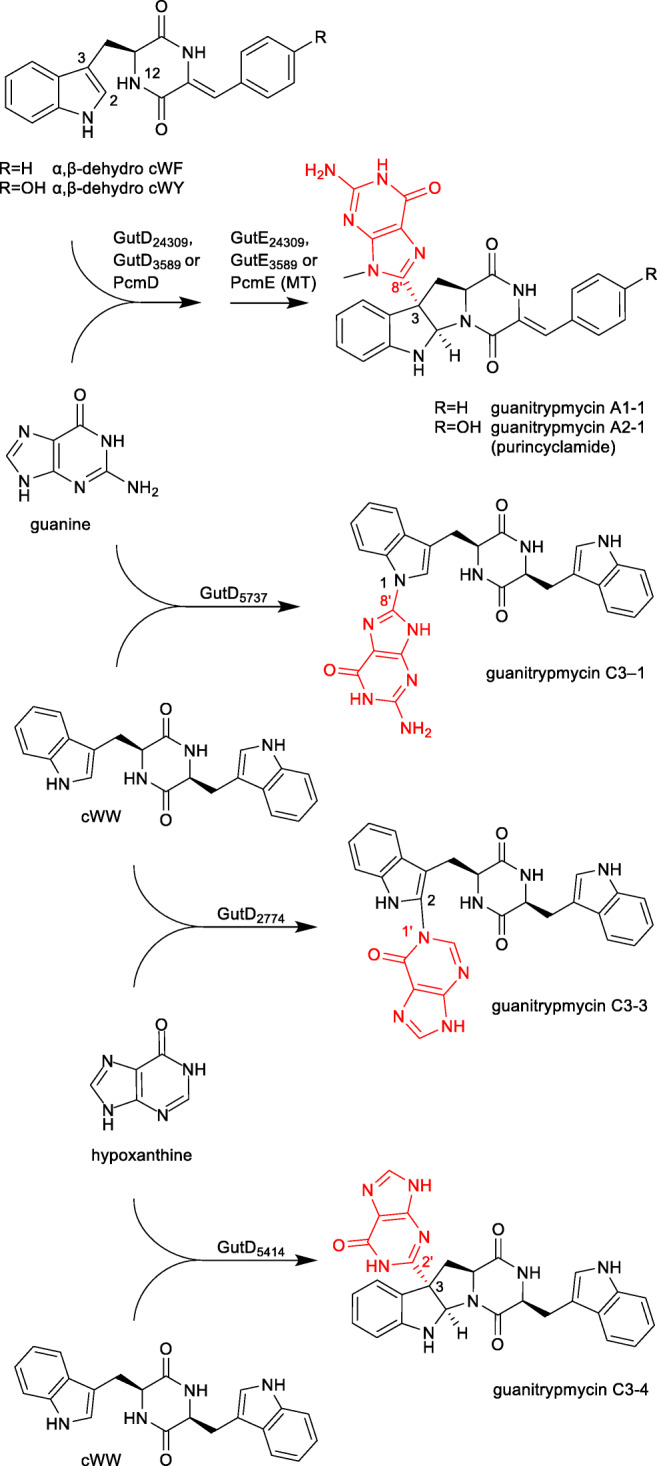
Coupling reactions of CDPs with nucleobases catalysed by P_450_s

Four biosynthetic pathways for guanitrypmycins bearing a guanine moieties have been reported so far (Fig. 6), including the two-gene cluster from *Streptomyces purpureus* NRRL B5737 with the P_450_ GutD_5737_ for coupling of guanine with cWW (Yu et al. 2018). Two very similar BGCs consisting of five genes were identified in *Streptomyces monomycini* NRRL B-24309 and *Streptomyces varsoviensis* NRRL B-3589. These genes code for four functional enzymes, *i.e.* CDPS, CDO encoded by two genes, P_450_ and MT. Both P_450_ enzymes, GutD_24309_ from strain B-24309 and GutD_3589_ from B-3589, catalyse the *C3*-guaninylation of dehydro CDP derivatives. Subsequent N-methylation at the guanine residue by GutE leads to the formation of cWY derivative guanitrypmycin A2-1. The BGC from strain B-3589 is also responsible for the formation of the cWF derivative guanitrypmycin A1-1 (Liu et al. 2019). An almost identical BGC for guanitrypmycin A2-1, termed purincyclamide in that paper, was later identified in *Streptomyces chrestomyceticus* NA4264, and the corresponding P_450_ enzyme was named PcmD (Shi et al. 2019).

Interestingly, the guanine is always attached *via* C8´ to either N1 or C3 position of the tryptophanyl residue. The first identified GutD_5737_ catalyses a C-N bond linkage, whereas other three enzymes, GutD_24309_, GutD_3589_ and PcmD, a C-C bond formation, followed by a cyclisation between C2 of the indole and N10 of the DKP rings.

In addition to the guanine transfer reactions, the CDPS-associated P_450_s can also catalyse the coupling of CDP with another nucleobase hypoxanthine. GutD_2774_ from *Streptomyces lavendulae* NRRL B-2774 and GutD_5414_ from *Streptomyces xanthophaeus* NRRL B-5414 use the same substrate cWW and attach hypoxanthine *via* its N1´ to C2 of the indole and C2´ to C3, respectively. The main final pathway products are identified correspondingly as guanitrypmycins C3-3 and C3-4 (see Fig. 6). GutD_2774_ and GutD_5414_ share sequence identities of 75 and 57% on the amino acid level with GutD_5737_ and were also found to be capable of using guanine as substrate, resulting in the formation of the minor side products guanitrypmycins C3-1 and C3-2, respectively (Yu et al. 2019). Although guanitrypmycins are unusual bacterial metabolites, no pharmacological and biological properties have been published yet, and their biological function remains therefore unknown. One important reason is their low solubility in aqueous milieu.

## P_450_s as DKP dimerases for C-C bond formation

**Fig. 7:**
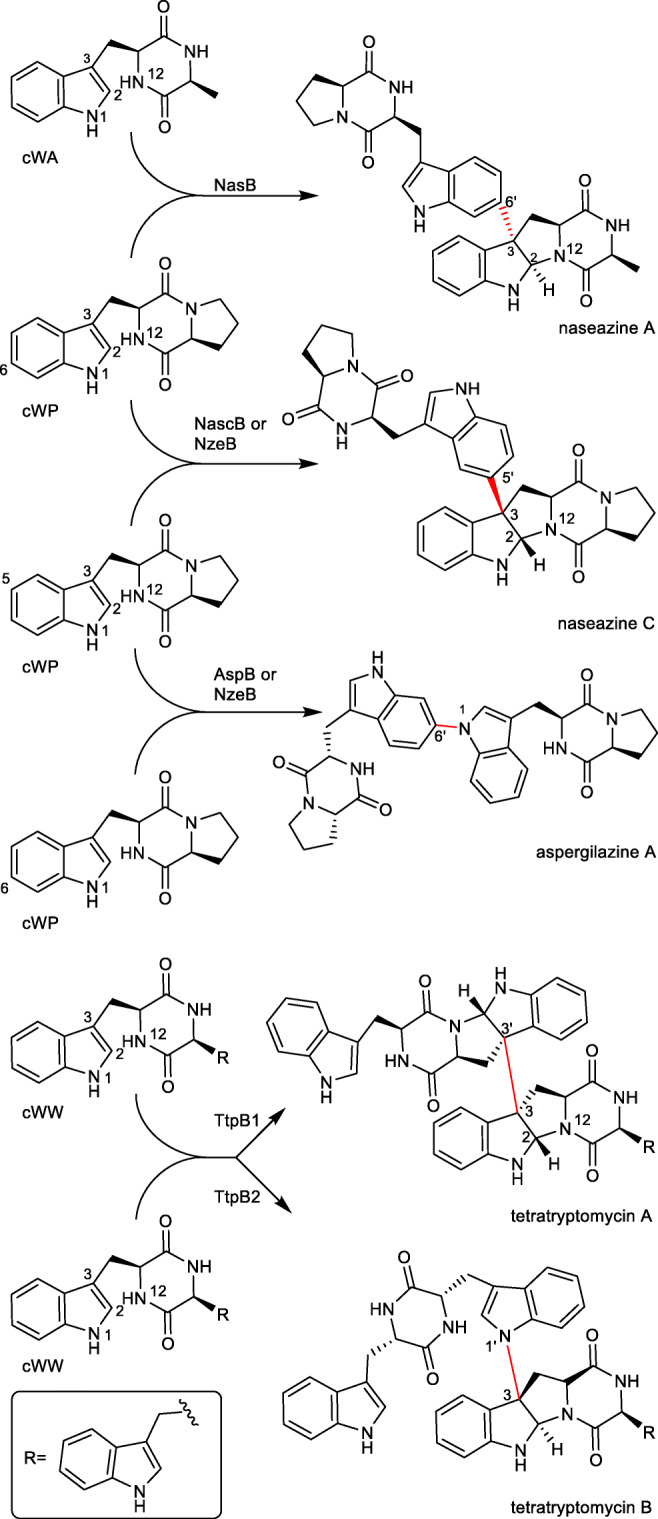
Dimerisation of CDPs catalysed by P_450_s

At least four CDPS-associated P_450_s catalyse dimerisation of tryptophan-containing CDPs *via* an intermolecular C-C bond formation between the two tryptophanyl moieties (Fig. 7). Their products are connected *via* C3 of one tryptophanyl moiety, accompanied by a cyclisation between C2 and N12 based on a Mannich reaction to form a pyrroloindoline system, in analogy to guanitrypmycins mentioned above (Tian et al. 2018).

Among the characterised CDP dimerases, NasB from *Streptomyces* sp. NRRL S-1868 uses cWP and cWA as substrates, resulting in the formation of naseazine A with a C3-C6´ linkage as the mere product (Yu and Li 2019). NascB from *Streptomyces* sp. CMB-MQ030 (Tian et al. 2018) and NzeB from *Streptomyces* sp. NRRL F-5053 (Shende et al. 2020) are cWP dimerases for C3-C5´ coupling with naseazine C as the main product. Two two-gene operons from *Saccharopolyspora antimicrobica* DSM 45119 are responsible for the formation of tetratryptomycins with four tryptophanyl units. TtpB1 catalyses C3-C3´connection between two cWW molecules (Liu et al. 2020). *In vitro* bioactivity testes revealed that naseazine C showsactivity against chloroquine-sensitive malaria parasites and its derivatives have protective activity against glutamate-induced PC-12 damage (Tian et al. 2018).

## P_450_s as DKP dimerases for C-N bond formation

The aforementioned NzeB also catalyses the N1-C6´dimerisation of two cWP molecules, resulting in the formation of aspergilazine A as minor side product (Shende et al. 2020). Aspergilazine A was identified as the main product of a two-gene BGC from *Streptomyces* sp. NRRL S-1868, with AspB as the responsible dimerase (Yu and Li 2019).

The second two-gene operon from *Saccharopolyspora antimicrobica* DSM 45119 is responsible for the formation of tetratryptomycin B. The P450 TtpB2 catalyses dimerisation of cWW *via* C3-N1´ coupling (see Fig. 7) (Liu et al. 2020). Tetratryptomycin B shows no antibacterial effects on cell lines of *E. coli*, *Bacillus subtilis*, *Staphylococcus aureus* or *Pseudomonas aeruginosa*.

## Structural basis and reaction mechanisms

The first crystal structure of the CDPS-associated P_450_s CYP121 from *Mycobacterium tuberculosis* has been solved in 2009 (Belin et al. 2009). Based on the structure together with QM/MM studies, a reaction mechanism with involvement of two radicals was proposed (Dumas et al. 2014). One tyrosyl residue is bound closely to the haem centre in proximity to the key oxidant species of P_450_s, whereas the other tyrosyl moiety points to the protein surface. The important intermediates bear unpaired electrons at *ortho*-position to the hydroxyl groups of both phenyl moieties. Intramolecular connection of the two radicals leads to the formation of mycocyclosin (Fig. 5) (Dumas et al. 2014).

**Fig. 8:**
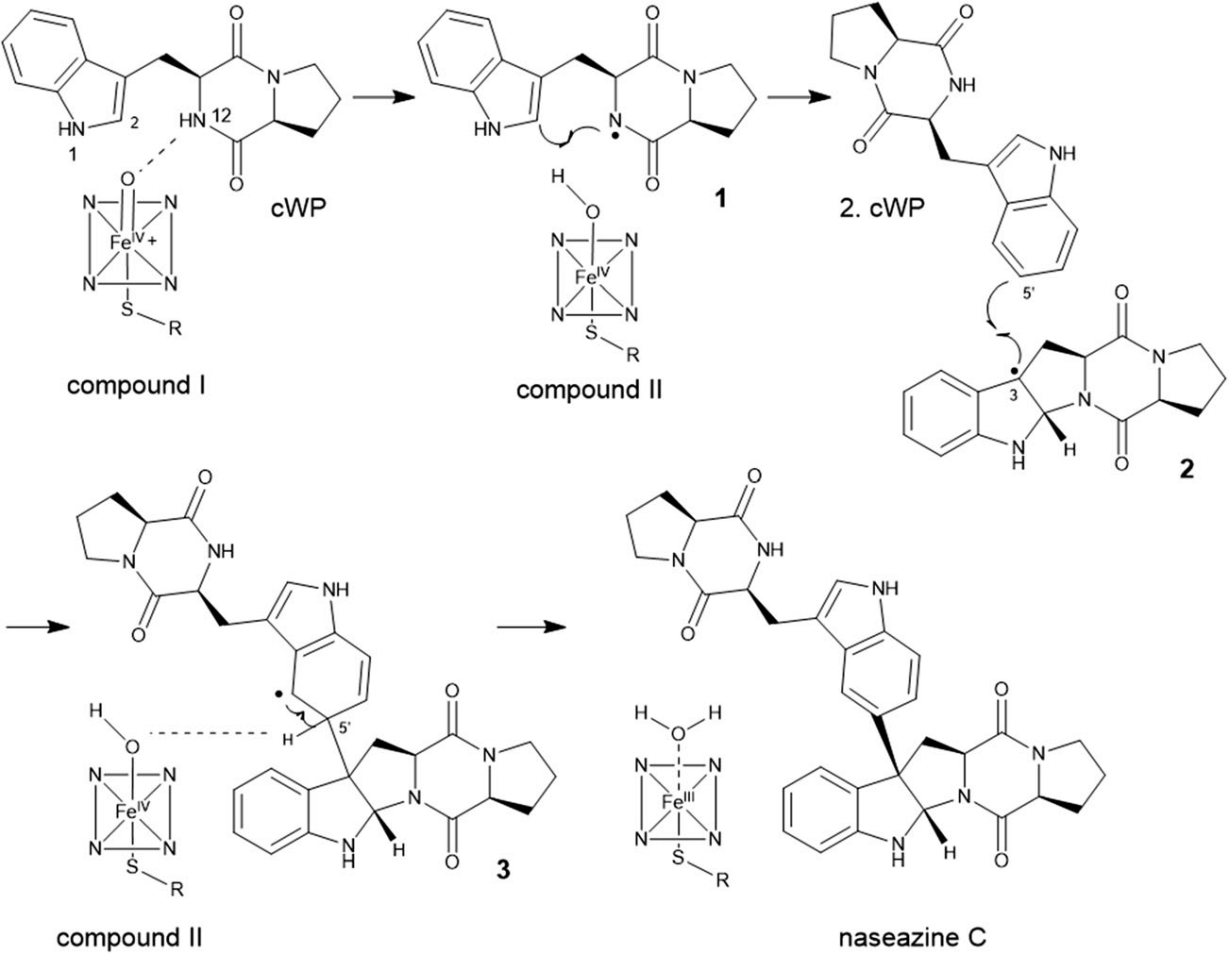
Proposed mechanism for NzeB reaction, modified from Shende et al. (2020)

Very recently, two groups published the structure of the same P_450_ from *Streptomyces* sp. NRRL F-5053, termed NzeB and Nas_F5053_, respectively (Shende et al. 2020; Sun et al. 2020). It was proposed that the dimerisation of cWP catalysed by this enzyme would also be *via* a radical-mediated mechanism (Fig. 8) (Shende et al. 2020). In contrast to CYP121, this dimerase only forms one radical at nitrogen N1 (Sun et al. 2020) or N12 (Shende et al. 2020) after abstraction of one hydrogen by compound I (intermediate 1). In the mechanism proposed by Shende et al. (2020), the resulting radical then shifts to C3 after cyclisation between N12 and C2 (intermediate 2). The C3 radical attacks subsequently C5’ of the tryptophanyl moiety of the second CDP (intermediate 3), followed by a re-aromatisation *via* elimination of the C5’-hydrogen mediated by compound II, resulting in naseazine C. Regarding the intermolecular C-N bond formation, a similar radical-mediated mechanism is strongly favoured (Shende et al. 2020). In contrast to the C-C dimerisation, the first hydrogen is abstracted from N1 instead of N12. The subsequent steps take place in analogy to those of C-C bond formation.

Sequence alignments of NascB, NzeB (synonym Nas_F5053_) and Nas_S1868_ (synonym AspB) revealed four critical amino acid residues in these P_450_s controlling the regio- and stereoselectivity. Mutation on the key residues at positions 65, 86, 284 and 288 led to the alteration of the regio- and stereospecificity of these enzymes (Sun et al. 2020).

## Conclusion

In this review, we summarised the intriguing reactions catalysed by bacterial CDPS-associated P_450_s like DKP ring aromatisation, CDP dimerisation and nucleobase transfer reactions. These P_450_s have the advantages that they are soluble proteins and can be easily overproduced in *E. coli*. Some of them show a flexible substrate tolerance towards CDP analogues (Tian et al. 2018), which could be used for production of designed CDP derivatives by chemoenzymatic and synthetic biological approaches, *e.g.* by recombination of different genes for CDPS, P_450_s or for other enzymes like prenyltransferases (Dubois et al. 2019).

Genome sequencing revealed the presence of a large number of *cdps*-containing BGCs including those with P_450_s as tailoring enzymes. More than 700 of such BGCs were identified in 93107 prokaryotic genomes (Skinnider et al. 2018). The number of the clusters of interest will undoubtedly increase with the number of ongoing sequencing projects. Targeted gene activation might not only uncover novel natural products but also reveal new functions of tailoring enzymes including P_450_s.
